# Toward a clinical practice guide in pharmacogenomics testing for functional polymorphisms of drug-metabolizing enzymes. Gene/drug pairs and barriers perceived in Spain

**DOI:** 10.3389/fgene.2012.00273

**Published:** 2012-11-26

**Authors:** José A. G. Agúndez, Francisco Abad-Santos, Ana Aldea, Hortensia Alonso-Navarro, María L. Bernal, Alberto M. Borobia, Emma Borrás, Miguel Carballo, Alfonso Carvajal, José D. García-Muñiz, Guillermo Gervasini, Félix J. Jiménez-Jiménez, María I. Lucena, Carmen Martínez, José A. Sacristán, Inés Salado, Blanca Sinués, Jorge Vicente, Elena García-Martín

**Affiliations:** ^1^Department of Pharmacology, University of ExtremaduraCáceres, Spain; ^2^Instituto de Salud Carlos III Spanish Research Network of Adverse Reactions to Allergens and DrugsMadrid, Spain; ^3^Servicio de Farmacologia Clínica, Hospital Universitario de la Princesa, Instituto de Investigación Sanitaria PrincesaMadrid, Spain; ^4^Unidad de Ensayos Clínicos, Hospital Universitario de CanariasLa Laguna, Spain; ^5^Department of Medicine-Neurology, Hospital Príncipe de Asturias, Alcalá de HenaresSpain; ^6^Section of Neurology, Hospital Universitario del Sureste, Arganda del ReySpain; ^7^Department of Pharmacology, University of ZaragozaZaragoza, Spain; ^8^Unidad de Farmacogenética Clínica, Servicio de Farmacología Clínica, Hospital Universitario La Paz de Madrid, Instituto de Investigación Hospital Universitario La Paz, Facultad de Medicina, Universidad Autónoma de MadridMadrid, Spain; ^9^Unitat de Genètica Molecular, Hospital de TerrassaTerrassa, Spain; ^10^Department of Pharmacology, University of ValladolidValladolid, Spain; ^11^Hospital Universitario de CeutaCeuta, Spain; ^12^Servicio de Farmacología Clínica, Hospital Universitario Virgen de la Victoria, Instituto de Investigación Biomédica de Málaga, Universidad de MálagaMálaga, Spain; ^13^Centro de Investigación Biomédica en Red de Enfermedades Hepáticas y DigestivasBarcelona, Spain; ^14^Department of Pharmacology, University of ExtremaduraBadajoz, Spain; ^15^Departamento Médico, Lilly S.A., AlcobendasMadrid, Spain; ^16^Centro de Estudios sobre la Seguridad de los Medicamentos, Universidad de ValladolidValladolid, Spain; ^17^Department of Biochemistry and Molecular Biology, University of ExtremaduraCáceres, Spain

**Keywords:** biomarkers, adverse drug reactions, pharmacogenomics, clinical recommendations, clinical relevance

## Abstract

The development of clinical practice recommendations or guidelines for the clinical use of biomarkers is an issue of great importance with regard to adverse drug reactions. The potential of pharmacogenomic biomarkers has been extensively investigated in recent years. However, several barriers to implementing the use of pharmacogenomics testing exist. We conducted a survey among members of the Spanish Societies of Pharmacology and Clinical Pharmacology to obtain information about the perception of such barriers and to compare the perceptions of participants about the relative importance of major gene/drug pairs. Of 11 potential barriers, the highest importance was attributed to lack of institutional support for pharmacogenomics testing, and to the issues related to the lack of guidelines. Of the proposed gene/drug pairs the highest importance was assigned to HLA-B/abacavir, UGT1A1/irinotecan, and CYP2D6/tamoxifen. In this perspective article, we compare the relative importance of 29 gene/drug pairs in the Spanish study with that of the same pairs in the American Society for Clinical Pharmacology and Therapeutics study, and we provide suggestions and areas of focus to develop a guide for clinical practice in pharmacogenomics testing.

Functional polymorphisms of drug-metabolizing enzymes are a major factor involved in adverse drug reactions. The development of pharmacogenomic biomarkers has evolved in recent years, mainly in a frame where these biomarkers are intended to be used as outcome biomarkers, that is, to substitute for a clinical outcome or predict an outcome of a disease or toxicity following treatment. While the most conservative pharmacogenomics views aim to stratify patient populations (patient selection biomarkers) into those who should or should not receive a given drug (Green and Guyer, 2011), other guidelines are intended to adjust drug dose based on pharmacogenomics tests (see for instance Swen et al., 2011). The limitations of pharmacogenomics-based dose adjustment are analyzed elsewhere (Agúndez et al., 2012) and will not be discussed here, but it should emphasized that part of the disenchantment experienced with pharmacogenomics in recent years is related to the overoptimistic expectation of making a safe and reliable personalized dose adjustment based on pharmacogenomics tests. Today we know that interindividual variability in drug metabolism and response exists, even within individuals with identical pharmacogenomics genotypes, and so pharmacogenomics is simply another factor to be considered in dose adjustment.

But in even the most conservative approaches to pharmacogenomics (whether individuals would respond to a determined drug, or what patient's odds are to experience adverse effects with a determined drug), many factors hamper rapid development of the clinical use of pharmacogenomics tests. These factors have been analyzed in detail elsewhere (Deverka et al., 2007; Haga and Burke, 2008; Agúndez, 2009; Agúndez et al., 2009, 2012; Relling et al., 2010; Matheis et al., 2011; Relling and Klein, 2011), but the relative importance of these factors has received little attention. In the frame of the Carlos III Institute of Health (ISCIII) Spanish Research Network of Adverse Reactions to Allergens and Drugs, we conducted a survey in 2012 among members of the Spanish Societies of Pharmacology and Clinical Pharmacology, together with other Spanish clinicians and geneticists closely related to pharmacogenomics, on the most important challenges to clinical implementation of pharmacogenomic tests. The profile of the participants was as follows: 59% were practicing physicians, 21% had an academic research profile, 16% were clinical laboratory professionals, and 5% worked in the pharmaceutical industry. The survey in Spain was designed to include the same gene/drug pairs and the same evaluation criteria as in a US survey (Relling and Klein, 2011) to obtain results which could be directly compared. In addition, we included a survey regarding potential barriers to implementing the use of pharmacogenomics testing (Agúndez et al., 2012). The responses were closed, ranking from 1 to 10 for every item. The ranks obtained for gene/drug pairs were re-scaled in a scale from 1 to 5 to make results comparable to those from the US survey (Relling and Klein, 2011).

**Figure 1** summarizes the results, which indicated three major groups of barriers. The most relevant group was related to low institutional promotion, and the second corresponded to lack of clinical guidelines, protocols, and other factors related to validity of pharmacogenetics tests. The barriers in this second group are closely related to one another and, in our opinion, are a major cause of lack of promotion and support for healthcare systems. A third group of barriers with less perceived importance in the survey was related to economical and institutional issues, knowledge of the pharmacogenomic background of the Spanish population and ethical, legal, or social implications.

**FIGURE 1:**
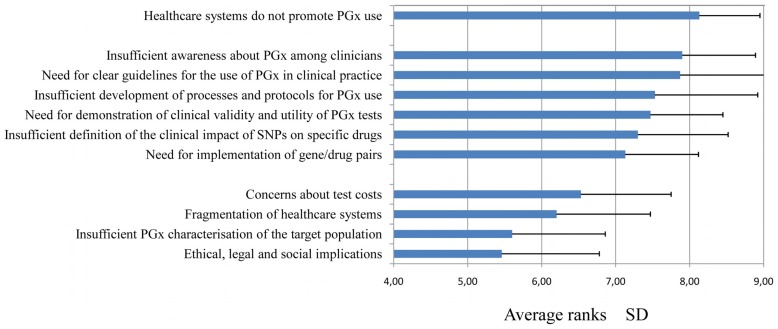
**Highest-ranking barriers to implementing the use of pharmacogenomics testing, based on a survey of Spanish Societies for Pharmacology and Clinical Pharmacology members in 2012.** Data related to average importance (on a scale of 1–10) are plotted along the *x*-axis.

Because most of the main barriers were related to lack of clinical guidelines and protocols, we included in our survey the same 29 gene/drug pairings listed in a previous study carried out among members of the Clinical Pharmacogenetics Implementation Consortium (CPIC; see http://www.pharmgkb.org/page/cpic) and members of the American Society for Clinical Pharmacology and Therapeutics (Relling and Klein, 2011). The highest ranked pairs (based on the perceived importance of the data linking the drug to the gene variation) were HLA-B/abacavir, UGT1A1/irinotecan, and CYP2D6/tamoxifen (**Figure 2**). While most gene/drugs pairs received a similar rank in both studies, the comparison revealed that in the Spanish study the gene pairs CYP2C9/celecoxib and CYP2D6/nortriptyline had higher ranks than in the US study, whereas the pairs CYP2C19/clopidogrel, GP6D/chloroquine, and both genes related to warfarin were ranked lower. Nevertheless, looking at the whole picture, the ranks are similar for most gene/drug pairs, thus indicating that guidelines for gene/drug pairs such as those which are currently being elaborated by CPIC members seem to be well suited for Spain, and probably for other European countries with a similar genetic background. The few discrepancies observed in **Figure 2** may be related to the differences in the pattern of drug use in the US and in Spain. CPIC guidelines on some of the gene/drug pairs mentioned have already been published (see for instance (Johnson et al., 2011; Crews et al., 2012; Wilke et al., 2012) and many others are currently underway (see http://www.pharmgkb.org/page/cpicGeneDrugPairs). Hopefully we will soon have guidelines available for all relevant gene/drug pairs.

**FIGURE 2:**
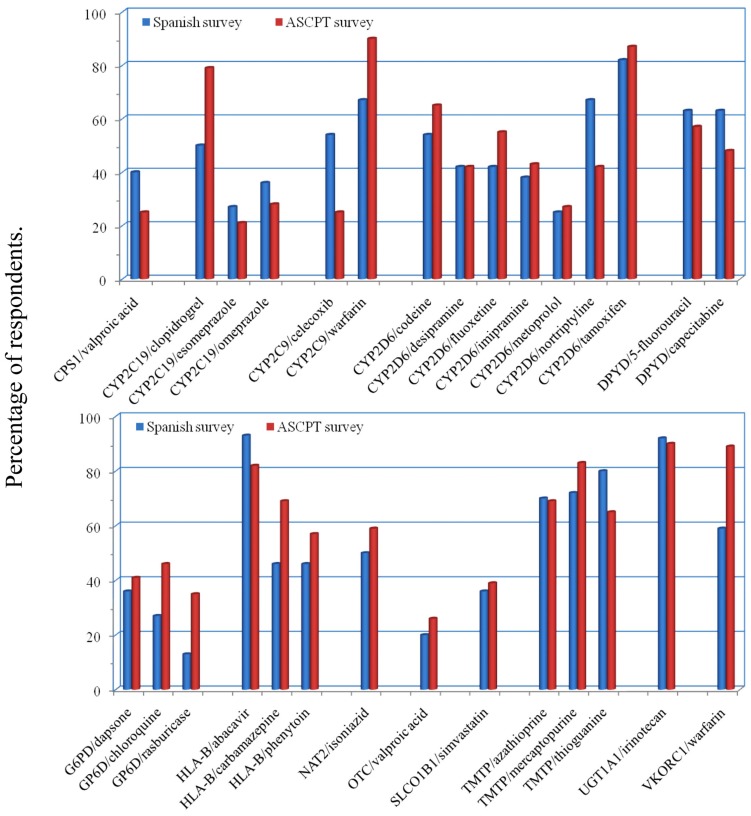
**Highest-ranking gene/drug pairs, based on a survey of Spanish Societies for Pharmacology and Clinical Pharmacology members in 2012 (blue bars), compared to a published survey of American Society for Clinical Pharmacology and Therapeutics (ASCPT) members conducted by CPIC (red bars).** Data related to the percentages of respondents who ranked the gene/drug pairs as 1 or 2 (on a scale of 1–5) are plotted along the *y*-axis.

Nevertheless, some remaining questions related to interethnic and intraethnic genetic variability (Garcia-Martin et al., 2006; Garcia-Martin, 2008; Borobia et al., 2009; Restrepo et al., 2011), refinement in phenotype inference (Agúndez et al., 2008; Ruiz et al., 2012), and detailed studies on the contribution of additional factors which may modify the strength of the association of gene/drugs pairs need to be dealt with. These studies should include gene expression and regulation, patient's metabolic profile with a given drug, and relevant lifestyle and environmental factors that influence drug metabolism or drug response. Once guidelines and protocols are ready, the major groups of barriers shown in **Figure 1** will be weakened. And, in consequence, this will hopefully lessen the impact of the first and most determinant barrier, since we believe that institutional support and promotion for the use of pharmacogenomics biomarkers will greatly improve as the influence of the other barriers decline.

## Conflict of Interest Statement

The authors declare that the research was conducted in the absence of any commercial or financial relationships that could be construed as a potential conflict of interest.
